# Sex differences in childhood cancer risk following ART conception: a registry-based study

**DOI:** 10.1093/humrep/deae285

**Published:** 2024-12-26

**Authors:** L L Oakley, D Kristjansson, M C Munthe-Kaas, H T Nguyen, Y Lee, H I Hanevik, L B Romundstad, R Lyle, S E Håberg

**Affiliations:** Centre for Fertility and Health, Norwegian Institute of Public Health, Oslo, Norway; Non-Communicable Disease Epidemiology Unit, London School of Hygiene and Tropical Medicine, London, UK; Centre for Fertility and Health, Norwegian Institute of Public Health, Oslo, Norway; Department of Genetics and Bioinformatics, Norwegian Institute of Public Health, Oslo, Norway; Centre for Fertility and Health, Norwegian Institute of Public Health, Oslo, Norway; Department of Paediatric Oncology and Haematology, Oslo University Hospital, Oslo, Norway; Centre for Fertility and Health, Norwegian Institute of Public Health, Oslo, Norway; Department of Community Medicine and Global Health, University of Oslo, Oslo, Norway; Centre for Fertility and Health, Norwegian Institute of Public Health, Oslo, Norway; Centre for Fertility and Health, Norwegian Institute of Public Health, Oslo, Norway; Department of Fertility, Telemark Hospital Trust, Skien, Norway; Centre for Fertility and Health, Norwegian Institute of Public Health, Oslo, Norway; Spiren Fertility Clinic, Trondheim, Norway; Centre for Fertility and Health, Norwegian Institute of Public Health, Oslo, Norway; Department of Medical Genetics, Oslo University Hospital, Oslo, Norway; Centre for Fertility and Health, Norwegian Institute of Public Health, Oslo, Norway; Department of Global Public Health and Primary Care, University of Bergen, Bergen, Norway

**Keywords:** cancer, cancer risk, childhood cancer, leukaemia, assisted reproductive technologies, ART, sex differences, Norway

## Abstract

**STUDY QUESTION:**

Does the risk of childhood cancer following ARTs vary by sex?

**SUMMARY ANSWER:**

In this registry-based study, some childhood cancers showed positive sex- and age-specific associations in children conceived using certain ART modalities, which were not evident in overall combined analyses.

**WHAT IS KNOWN ALREADY:**

The relationship between ART and risk of childhood cancer has shown diverse outcomes in prior research. Studies examining whether there are sex differences in childhood cancer risk after ART conception are lacking.

**STUDY DESIGN, SIZE, DURATION:**

This registry-based cohort study included all children born in Norway between 1984 and 2022 (n = 2 255 025), followed until 31 December 2023.

**PARTICIPANTS/MATERIALS, SETTING, METHODS:**

Children were identified via the Medical Birth Registry of Norway, and information was extracted on whether they were conceived via ART (defined as IVF/ICSI). Of the 2 255 025 children included in the study, 53 694 were ART-conceived. Birth records were linked to the Cancer Registry of Norway. Childhood cancer was defined as a cancer diagnosis according to the International Classification of Childhood Cancer Third Edition (ICCC-3) before the age of 18 years. Cox regression models were used to estimate the age- and sex-specific risk of cancer for ART-conceived children compared to children not conceived via ART.

**MAIN RESULTS AND THE ROLE OF CHANCE:**

Among all children, 0.25% had a cancer diagnosis before the age of 18 years. The cumulative incidence of cancer was higher in children conceived by ART (IVF/ICSI) than in those not conceived via ART (21.5 vs 17.5 per 100 000 person-years, *P *=* *0.04), and especially higher in boys conceived with ICSI or after cryopreserved embryo transfer. When combining all age groups, both sexes and all cancer types, there was little evidence of increased cancer risk with ART (adjusted hazard ratio (aHR) 1.13, 95% CI 0.94–1.36). However, differences were found when stratifying by age and sex. From age 5–9 years, ART-conceived children had a higher overall risk of cancer (aHR 1.53, 95% CI 1.06–2.20), with a slightly higher estimate in boys (aHR 1.73, 95% CI 1.09–2.74), than in girls (aHR 1.28, 95% CI 0.70–2.33). The risk was not higher up to age 5 years, or after age 10 years. In combined analyses, there was no overall increased risk after ICSI. When stratifying by sex, a higher risk was seen after ICSI for boys (aHR 1.69, 95% CI 1.18–2.42), but not for girls (aHR 0.65, 95% CI 0.37–1.16). The combined risk after cryopreservation (aHR 1.42, 95% CI 0.95–2.13) was driven by a higher risk in boys (aHR 1.79, 95% CI 1.09–2.94), while no evidence of an association was found in girls (aHR 1.01, 95% CI 0.50–2.03). No increased risk was seen with IVF or after fresh transfer for either boys or girls.

**LIMITATIONS, REASONS FOR CAUTION:**

Childhood cancer is a rare outcome, and some analyses of cancer subtypes were likely underpowered.

**WIDER IMPLICATIONS OF THE FINDINGS:**

Results from this large registry-based study suggest that addressing age- and sex-specific differences in the risk of childhood cancer following ART conception reveals increased risks for certain groups. Our findings require further study with consideration of possible underlying sex-specific mechanisms related to ART and different childhood cancers.

**STUDY FUNDING/COMPETING INTEREST(S):**

This work was funded by: the Research Council of Norway through its Centres of Excellence Funding Scheme (project number 262700); the Norwegian Cancer Association (project number 244291); and the Norwegian Institute of Public Health. The funding agencies had no role in the conceptualization, design, data collection, analysis, decision to publish, or preparation of the manuscript. The authors declare no conflict of interests.

**TRIAL REGISTRATION NUMBER:**

N/A.

## Introduction

ART, which includes IVF and ICSI, is a medical treatment offered to couples who have difficulty conceiving naturally. To date, more than 10 million ART-conceived children have been born (De Geyter *et al.*, 2020; Adamson *et al.*, 2023). ART techniques, which include the pharmacological stimulation of ovarian follicles, oocyte retrieval and sperm preparation, and culture and cryopreservation of embryos, differ substantially from natural conception. Concerns have been raised regarding the potential long-term health risks of children conceived through ART (Chen and Heilbronn, 2017; Berntsen *et al.*, 2019; Sciorio *et al.*, 2023). In particular, the relationship between ART and risk of childhood cancer has shown diverse outcomes in prior research (Hargreave *et al.*, 2013, 2019; Lerner-Geva *et al.*, 2017; Williams *et al.*, 2018; Spector *et al.*, 2019; Sargisian *et al.*, 2022; Weng *et al.*, 2022), and whether children born after ART are at increased risk of childhood cancer remains unclear.

Several studies worldwide of children born after ART have reported no overall increased risk of cancer for children conceived after ART (Raimondi *et al.*, 2005; Sundh *et al.*, 2014; Gilboa *et al.*, 2019; Hargreave *et al.*, 2019; Spaan *et al.*, 2019; Spector *et al.*, 2019). However, childhood cancers originate in different tissues with different aetiologies. Multiple studies have indicated a heightened risk of specific types of childhood cancers following ART. Several studies have found a higher rate of hepatic tumours among children conceived through ART (Hargreave *et al.*, 2013; Williams *et al.*, 2013, 2018; Spector *et al.*, 2019; Weng *et al.*, 2022). Also, the risks of central nervous system (CNS) tumours (Sundh *et al.*, 2014), including neuroblastomas (Hargreave *et al.*, 2013), and the risks of retinoblastomas (Hargreave *et al.*, 2013; Lerner-Geva *et al.*, 2017) and leukaemia (Reigstad *et al.*, 2016; Weng *et al.*, 2022) have been found to be higher in children conceived with ART. Similarly, malignant epithelial neoplasms (Sundh *et al.*, 2014), including melanoma (Sargisian *et al.*, 2022), have also been shown to be more frequent in children conceived with ART. There is a higher incidence of childhood cancers in boys compared to girls, and several of the subtypes above are more frequent in boys (Steliarova-Foucher *et al.*, 2017b). Still, studies examining whether there are sex differences in cancer risk after ART conception are lacking. We identified only one previous study that assessed cancer risk after mode of conception and stratified estimates by sex, with the authors concluding there was no evidence that risk differed by child sex (Weng *et al.*, 2022).

Some subtypes of childhood cancer risk are more commonly diagnosed in certain age periods during childhood. While solid and liquid malignancies are the most common in the youngest age group (0–5 years), and solid tumours again are the most common tumour in age 10–18 years, the CNS tumours are slightly more commonly diagnosed in the age group 5–10 years (Stensvold and Dahlen, 2024).

Risk of childhood cancer may also vary by ART treatment modality. In particular, previous studies have suggested that conceptions that include the transfer of cryopreserved embryos (Sargisian *et al.*, 2022) or fertilization by ICSI (Spaan *et al.*, 2019) may be associated with higher cancer risks than fresh embryo transfers or IVF without ICSI. However, whether there are any sex-specific risks with these different modalities has not yet been investigated.

While the biological mechanisms underlying associations between ART and cancer risk are unclear, an epigenetic study of ART offspring found 10 differentially methylated CpGs within the bidirectional promoter of *BRCA1* in children conceived with ART (Håberg *et al.*, 2022). *BRCA1* is involved in cell division and plays an important role in genome maintenance and gene expression (Tarsounas and Sung, 2020). *BRCA1* is established as a risk gene for several cancers in adult age, but alterations in *BRCA1* function may also influence risk of cancer in younger ages (Ford *et al.*, 1994). The risk of cancer with genetic alteration of *BRCA1* is higher among females compared to males, and *BRCA1*-related cancers in females have an earlier average age of diagnosis compared to *BRCA1*-related cancers in males (Thompson and Easton, 2002; Tai *et al.*, 2007).

Leveraging over 39 years of data from high-quality and comprehensive Norwegian registries, the aim of this study was to evaluate whether risk of childhood cancer in children conceived with ART differed by sex and age, and whether there were sex differences in risk of cancer by ART treatment method and childhood cancer type.

## Materials and methods

Records of all registered births in Norway between 1 January 1984 (the year of the first ART conception in Norway) and 31 December 2022 were extracted from the Medical Birth Registry of Norway (MBRN). Birth records were linked with the Cancer Registry of Norway and the Population Register using the unique person ID number allocated to all Norwegian citizens. We extracted cancer diagnoses to 31 December 2023. Further information on data sources is presented in Supplementary Table S1.

The established definition of ART includes only assisted conception methods which involve the manual handling of oocytes and sperm, or embryo outside the body (Zegers-Hochschild *et al.*, 2009). We therefore defined ART as only IVF or ICSI. The non-ART (non-IVF/ICSI) group contains births conceived by intrauterine insemination and ovulation induction. Births conceived by IVF/ICSI were to couples or single women with a diagnosis of infertility, and could include treatments with donor sperm. IVF/ICSI for preimplantation diagnosis was also included, but in a very small number (<0.1% of the IVF/ICSI cases). All clinics which provide ART treatment in Norway are mandated to notify the MBRN of any pregnancy resulting from ART. Information on whether the pregnancy was conceived by ART is also recorded on the birth notification form completed by midwives. These two sources of information within the MRBN were used to ascertain whether the birth resulted from ART conception.

All cancer diagnoses in the cancer registry are coded according to topography (site), morphology, and tumour behaviour using the International Classification of Diseases for Oncology Third Edition (ICD-0-3). To reflect the importance of morphology rather than site for cancers in childhood, all diagnoses were converted to the International Classification of Childhood Cancer Third Edition (ICCC-3) (Steliarova-Foucher *et al.*, 2017a) using ICD-0-3 codes. We defined childhood cancer as the presence of at least one ICCC-3 diagnosis before the age of 18 years.

### Statistical analyses

We used Cox proportional hazards regression to estimate the association between ART conception and childhood cancer, for boys and girls combined and also stratified by sex and age group. The time metric was attained age, with time at risk beginning at birth and ending at first cancer diagnosis, 18th birthday or 31 December 2023, whichever came first. Children were censored at death not due to cancer or emigration until December 2022, while these data were not available for 2023. The main outcome was any diagnosis of childhood cancer by attained age (<18 years; <5 years, 5 to <10 years, 10 to <18 years). The risk of childhood cancer was also assessed according to whether the specific ART method used was IVF only or ICSI, and whether the embryo transferred was fresh or cryopreserved-thawed. To take into account secular trends in ART methods, we conducted sensitivity analyses restricted to births from 2000 onwards to reflect the increasing use of ART over the last 25 years. Using the first diagnosis of cancer by ICCC-3 group, we also conducted childhood cancer-type specific analyses for subtypes with at least five children in the ART group.

All analyses were adjusted for birth year, maternal age, paternal age, parity, multiple birth, and parental history of cancer. Due to the possibility that multiple birth may operate as a mediator as well as potential confounder of the association under study, we also conducted a sensitivity analysis without adjustment for multiple birth. We used robust standard errors to account for dependency between siblings. In sensitivity analyses, we calculated effect estimates and bias-corrected confidence intervals based on statistics from 200 bootstrap samples using resampling without replacement (Efron and Tibshirani, 1986). The bootstrap method is highly effective for approximating the sampling distribution of statistics, especially for small sample sizes such as those observed for subtypes of childhood cancer in this analysis (Efron and Tibshirani, 1986; Stine, 1989). The validity of the proportional hazards assumption was assessed using Schoenfeld residuals, and there was no evidence of violation. Plots of scaled Schoenfeld residuals against time are presented in Supplementary Fig. S1. As the percentage of missing data was extremely low, no imputation was done for missing data. All analyses were performed using Stata version 17.

### Ethical approval

This study was approved by the Norwegian Regional Committee for Medicine and Health Research Ethics (No. 282989) which provided a waiver of consent for participants.

## Results

### Overall childhood cancer

There were 2 255 025 eligible children born between 1 January 1984 and 31 December 2022, of whom 2.4% (n = 53 694) were conceived by ART (IVF/ICSI) (Supplementary Fig. S2). The mean length of follow-up was 13.7 years (SD 5.8).

The mothers of children who were conceived via ART were more likely to be primiparous and older, and less likely to report smoking in pregnancy (Table 1, data not shown). While the birth years for children not conceived via ART were evenly distributed across birth years, children conceived by ART were concentrated in more recent birth years with 88% (n = 47 218) recorded from 2000 onwards. Compared to children not conceived via ART, those conceived via ART were more likely to be low birthweight, born preterm, and to be part of a multiple birth (Table 1).

**Table 1.** Characteristics of children born 1984–2022 in Norway (n = 2 255 025).

	Children born 1984–2022					
	All (N = 2 255 025)		ART-conceived (IVF/ICSI) (N = 53 694)		Non-ART conceived (N = 2 201 331)	
	n	(%)	n	(%)	n	(%)
**Year of birth**						
**1984–1993**	564 908	(25.1)	2099	(3.9)	562 809	(25.6)
**1994–2003**	585 574	(26.0)	9278	(17.3)	576 296	(26.2)
**2004–2013**	597 850	(26.5)	18 288	(34.1)	579 562	(26.3)
**2014–2022**	506 693	(22.5)	24 029	(44.8)	482 664	(21.9)
**Sex**						
**Male**	1 158 443	(51.4)	27 666	(51.5)	1 130 777	(51.4)
**Female**	1 096 582	(48.6)	26 028	(48.5)	1 070 554	(48.6)
**Gestation length** ^a^						
***Mean (days) (SD)***	*279 (14.6)*		*271 (19.8)*		*279 (14.4)*	
**<37 weeks**	139 252	(6.2)	8924	(16.6)	130 328	(5.9)
**≥37 weeks**	2 032 898	(90.1)	42 500	(79.2)	1 990 398	(90.4)
**Birthweight** ^b^						
***Mean (SD)***	*3512 (597)*		*3232 (750)*		*3519 (591)*	
**<2500 g**	105 126	(4.7)	7934	(14.8)	97 192	(4.4)
**≥2500 g**	2 148 347	(95.3)	45 730	(85.2)	2 102 617	(95.5)
**Multiplicity**						
**Singleton**	2 186 175	(96.9)	41 872	(78.0)	2 144 303	(97.4)
**Twin/higher order multiple**	68 850	(3.1)	11 822	(22.0)	41 872	(1.9)
**Maternal parity**						
**Primiparous**	946 894	(42.0)	33 627	(62.6)	913 267	(41.5)
**Multiparous**	1 308 131	(58.0)	20 067	(37.4)	1 288 064	(58.5)
**Maternal age at birth**						
***Mean (SD)***	*29.1 (5.2)*		*33.5 (4.35)*		*29.0 (5.17)*	
**<24**	439 997	(19.5)	762	(1.4)	439 235	(20.0)
**25–29**	767 541	(34.0)	9268	(17.3)	758 273	(34.4)
**30–34**	695 531	(30.8)	22 018	(41.0)	673 513	(30.6)
**35–39**	296 702	(13.2)	17 362	(32.3)	279 340	(12.7)
**≥40**	55 254	(2.5)	4284	(8.0)	50 970	(2.3)

*Note*: presented as mean (SD) or count (percentages).

a Missing data on gestation length n = 82 875.

b Missing data on birthweight n = 1552.

**Figure 1.**
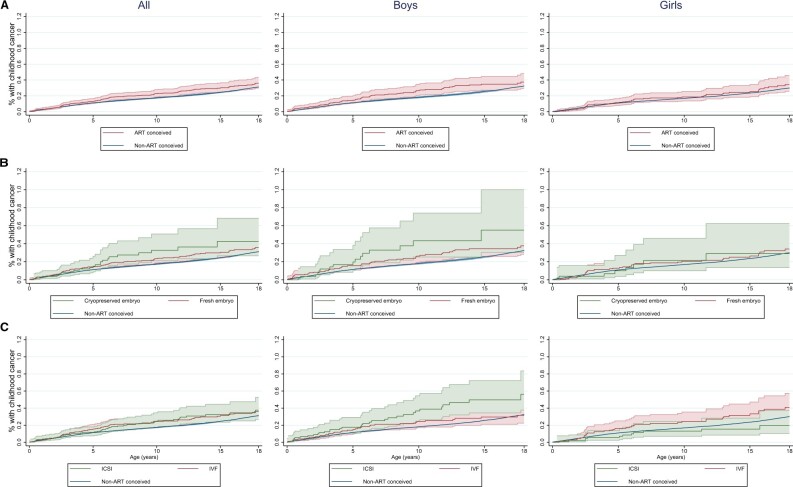
**Overall and sex-stratified cumulative incidence curves for childhood cancer.** (**A**) Any ART conception, (**B**) fresh versus cryopreserved embryos, (**C**) ICSI versus IVF. *P*-values for log-rank tests: any ART/non-ART: *P*=0.04 (all children), *P*=0.04 (boys), *P*=0.39 (girls); cryopreserved/fresh/non-ART: *P*=0.02 (all children), *P*=0.008 (boys), *P*=0.60 (girls); IVF/ICSI/non-ART: *P*=0.05 (all children), *P*=0.002 (boys), *P*=0.06 (girls).

Among the total study population, 5620 (0.25%) children had a record of at least one ICCC-3 diagnosis in the cancer registry before their 18th birthday. Of these 5620 children, 74 had more than one cancer diagnosis. Among ART-conceived children, there were 126 (0.23%) children with cancer, and among children not conceived via ART, there were 5494 (0.25%) with cancer. The cumulative incidence of childhood cancer among all children was higher in those conceived by ART compared to non-ART-conceived children (21.5 per 100 000 person-years for ART-conceived, 17.5 per 100 000 person-years for non-ART conceived; log-rank *P *=* *0.04; Fig. 1A). When combining both sexes, all cancers, and all ages from birth to age 18 years, there was no strong evidence of an overall increased risk in adjusted analysis (adjusted hazard ratio (aHR) 1.13, 95% CI 0.94, 1.36 based on 126 cases among ART-conceived children; Table 2).

**Table 2.** **Overall and sex-stratified association between ART conception (IVF/ICSI) and childhood cancer by age group**.

		All children			Boys			Girls		
Age		No. of cases	Hazard ratio		No. of cases	Hazard ratio		No. of cases	Hazard ratio	
			(95% CI)			(95% CI)			(95% CI)	
			Unadjusted	**Adjusted** ^a^		Unadjusted	**Adjusted** ^a^		Unadjusted	**Adjusted** ^a^
**0 to <18 years**	**ALL**	5620			3001			2619		
	**Non-ART**	5494	ref	ref	2930	ref	ref	2564	ref	ref
	**ART (IVF/ICSI)**	126	**1.20 (1.01, 1.44)**	1.13 (0.94, 1.36)	71	1.27 (1.01, 1.61)	1.22 (0.95, 1.57)	55	1.12 (0.86, 1.47)	1.03 (0.78, 1.37)
**0 to <5 years**	**ALL**	2433			1283			1150		
	**Non-ART**	2369	ref	ref	1246	ref	ref	1123	ref	ref
	**ART (IVF/ICSI)**	64	1.17 (0.91, 1.50)	1.09 (0.84, 1.42)	37	1.28 (0.92, 1.77)	1.23 (0.87, 1.74)	27	1.04 (0.71, 1.53)	0.95 (0.64, 1.41)
**≥5 to <10 years**	**ALL**	1190			649			541		
	**Non-ART**	1155	ref	ref	627	ref	ref	528	ref	ref
	**ART (IVF/ICSI)**	35	**1.59 (1.13, 2.22)**	**1.53 (1.06, 2.20)**	22	**1.84 (1.20, 2.81)**	**1.73 (1.09, 2.74)**	13	1.29 (0.74, 2.23)	1.28 (0.70, 2.33)
**≥10 to <18 years**	**ALL**	1997			1069			928		
	**Non-ART**	1970	ref	ref	1057	ref	ref	913	ref	ref
	**ART (IVF/ICSI)**	27	0.97 (0.67, 1.42)	0.91 (0.61, 1.34)	12	0.81 (0.46, 1.43)	0.79 (0.43, 1.44)	15	1.16 (0.70, 1.94)	1.02 (0.61, 1.71)

a Adjusted for birth year, maternal age, paternal age, multiple births, parity, and parental history of cancer. The reference level corresponds to non-ART.

Bold font indicates statistical significance (*P* < 0.05).

### Sex- and age-stratified cancer risk

A trend towards a higher cumulative incidence of childhood cancer in ART-conceived children was seen in boys but there was no clear indication of increased incidence in girls (*P*_boys_ = 0.04, *P*_girls_=0.39; Fig. 1A). When combining all ages and all cancers, there was no clear evidence of an overall association between ART conception and childhood cancer before age 18 years in either boys or girls (aHR for boys 1.22, 95% CI 0.95, 1.57, based on 71 cases among ART-conceived boys; aHR for girls 1.03, 95% CI 0.78, 1.37, based on 55 cases among ART-conceived girls; Table 2). There was little evidence of increased risk of childhood cancer following ART in the youngest (0 to <5 years) or oldest (10 to <18 years) age groups. However, among children aged 5–9 years there was evidence of an increased risk of cancer in ART-conceived boys (aHR 1.73, 95% CI 1.09, 2.74, based on 22 cases among ART-conceived boys), but no strong evidence of increased risk among ART-conceived girls (aHR 1.28, 95% CI 0.70, 2.33, based on 13 cases among ART-conceived girls).

### Childhood cancer and ART method

Among children conceived by ART, just over half (53.7%, n = 28 851) were conceived by IVF, one-third by ICSI (34.6%, n = 18 588), and the remainder were registered with either unknown or a combination of methods (11.7%, n = 6255) (Supplementary Table S2).

When analysing girls and boys up to age 18 years together, there was no strong evidence that either IVF or ICSI were associated with a higher risk of childhood cancer (IVF aHR 1.18, 95% CI 0.93, 1.49, based on 74 cases among IVF-conceived; ICSI aHR 1.18, 95% CI 0.87, 1.59, based on 44 cases among ICSI-conceived; Fig. 1C, Table 3). However, when stratifying by sex, boys conceived via ICSI had a higher risk of childhood cancer compared to boys not conceived via ART (aHR 1.69, 95% CI 1.18, 2.42, based on 32 cases among ICSI-conceived boys), while no increased risk was seen in girls conceived by ICSI (aHR 0.65, 95% CI 0.37, 1.16, based on 12 cases among ICSI-conceived girls). With IVF, no clear increased risk of cancer was seen in boys or girls (aHR 1.07, 95% CI 0.77, 1.51, based on 36 IVF-conceived boys; aHR 1.29, 95% CI 0.93, 1.80, based on 38 IVF-conceived girls; Table 3).

**Table 3.** Overall and sex-stratified association between ART conception (IVF/ICSI) and childhood cancer by ART method.

		All children			Boys			Girls		
		No. of cases	Hazard ratio		No. of cases	Hazard ratio		No. of cases	Hazard ratio	
			(95% CI)			(95% CI)			(95% CI)	
			Unadjusted	**Adjusted** ^a^		Unadjusted	**Adjusted** ^a^		Unadjusted	**Adjusted** ^a^
**ART method**	**Non-ART**	5494	ref	ref	2930	ref	ref	2564	ref	ref
	**IVF**	74	1.23 (0.98, 1.55)	1.18 (0.93, 1.49)	36	1.10 (0.79, 1.52)	1.07 (0.77, 1.51)	38	**1.39 (1.01, 1.92)**	1.29 (0.93, 1.80)
	**ICSI**	44	1.28 (0.95, 1.72)	1.18 (0.87, 1.59)	32	**1.82 (1.29, 2.58)**	**1.69 (1.18, 2.42)**	12	0.72 (0.41, 1.26)	0.65 (0.37, 1.16)
**Embryo type**	**Non-ART**	5494	ref	ref	2930	ref	ref	2564	ref	ref
	**Fresh embryo**	92	1.22 (0.99, 1.50)	1.15 (0.93, 1.43)	51	1.27 (0.96, 1.67)	1.22 (0.91, 1.63)	41	1.16 (0.85, 1.58)	1.08 (0.78, 1.48)
	**Cryopreserved embryo**	24	**1.55 (1.04, 2.32)**	1.42 (0.95, 2.13)	**16**	**1.92 (1.17, 3.14)**	**1.79 (1.09, 2.94)**	8	1.12 (0.56, 2.24)	1.01 (0.50, 2.03)

a Adjusted for birth year, maternal age, paternal age, multiple births, parity, and parental history of cancer. The reference level corresponds to non-ART.

Bold font indicates statistical significance (*P* < 0.05).

There was some (not significant) evidence that conception following the transfer of cryopreserved embryos was associated with a higher risk of childhood cancer before age 18 years (aHR 1.42, 95 CI 0.95, 2.13, based on 24 cases among those conceived via the use of cryopreserved embryo) but the risk was not increased following fresh embryo transfer (aHR 1.15, 95% 0.93, 1.43, based on 92 cases among children conceived via fresh embryo transfer; Fig. 1B, Table 3). Compared to boys not conceived via ART, the use of cryopreserved embryos was associated with an increased cancer risk (aHR 1.79, 95% CI 1.09, 2.94, based on 16 cases among boys conceived via cryopreserved embryos), but there was no strong evidence of an increased risk for boys conceived by fresh embryo transfer (Table 3). Among girls, neither fresh embryo transfer nor the use of cryopreserved embryos were associated with the risk of childhood cancer (Table 3).

Estimates for the association between ART method and childhood cancer <18 years restricting to births after the year 2000 were similar to the estimates derived using the full sample (Supplementary Table S3). Adjusted estimates were also very similar regardless of whether multiple birth was adjusted for or not (Supplementary Table S4).

Bootstrap-estimated HRs and bias-corrected confidence intervals are presented in Supplementary Tables S5 and S6. Bootstrap estimates were very similar in both magnitude, directionality, and significance to the main estimates, supporting validity of the results obtained from Cox models.

### Risk of childhood cancer by cancer type

Additional analyses were carried out by childhood cancer types where there were more than five cases in the ART group (Supplementary Table S7). For leukaemia (ICCC-3 group 1), there was an increased risk following ART conception among boys (aHR 1.60, 95% CI 1.04, 2.46, based on 23 cases among ART-conceived boys), but no evidence of an increased risk among girls For lymphomas (ICCC-3 group II), there was a trend towards increased risk of childhood cancer before age 18 years, with similar estimates observed for boys and girls, though the evidence was not strong (aHR for boys 1.55, 95% CI 0.80, 3.01, based on 10 cases in ART-conceived boys; aHR for girls 1.65, 95% CI 0.77, 3.56, based on seven cases in ART-conceived girls). There was no evidence of an increased risk of cancer following ART conception for the following childhood cancer subtypes: AML, ALL, CNS tumours (group III), and neuroblastoma and other peripheral nervous cell tumours (group IV). For both renal tumours (ICCC-3 group VI) and soft tissue sarcomas (group IX), the aHR was suggestive of an increased risk for boys, though there was no clear evidence and numbers were small (aHR for renal tumours in boys 2.34, 9% CI 0.86, 6.38; aHR for soft tissue sarcomas in boys 2.16, 95% CI 0.93, 5.02; case numbers among ART-conceived children cannot be reported by sex for privacy reasons). The sample size was too small to carry out estimations among girls. There were insufficient cases to look at the following childhood cancer subtypes: retinoblastoma (group V), hepatic tumours (group VII), bone tumours (group VIII), germ cell and gonadal tumours (group X), epithelial tumours and melanoma (group XI), or other and unspecified tumours (group XII).

## Discussion

Using Norwegian registry data on over 2 million infants born between 1984 and 2020, we found that stratifying on age and sex is important when studying cancer risk in children conceived with ART. There was evidence of an increased risk of being diagnosed with childhood cancer following ART conception between 5 and 9 years, especially in boys. A higher risk of cancer was seen among boys of any age conceived via ICSI, while this was not seen in girls. The higher risk seen among all children after cryopreservation was driven by a higher risk in boys, while there was no strong evidence of association among girls.

A number of previous studies have investigated the association between ART conception and childhood cancer, with conflicting findings (Raimondi *et al.*, 2005; Sundh *et al.*, 2014; Reigstad *et al.*, 2016; Spaan *et al.*, 2019; Spector *et al.*, 2019; Sargisian *et al.*, 2022; Weng *et al.*, 2022). The present study includes more than twice as many ART-conceived children as the most recently previously published analysis of Norwegian data, with an additional 12 years of follow-up (Reigstad *et al.*, 2016). Although childhood cancers include various cancer type with different aetiologies, and differ with age and sex, studies investigating the age- or sex-stratified risk of childhood cancer following ART conception have been lacking.

Our results suggest that the risk of childhood cancer in offspring following ART may vary by offspring sex and age. Therefore, it would be prudent for future studies to avoid combining both sexes, all cancer types, and all childhood ages together when investigating the risk of childhood cancer after ART. While there was no overall association between ART conception and childhood cancer before age 18 years in either boys or girls, stratification by age revealed noteworthy patterns. Among children aged 5–9 years, there was evidence of an increased risk of cancer, especially in ART-conceived boys. It was unclear what type of cancer drove the higher risk in this age group. For girls in this age group, although not statistically significant, there was a trend towards an increased risk at age 5–9 years. These findings underscore the importance of considering age-specific risks when assessing the impact of ART conception on childhood cancer.

We also found that there were sex-specific risks of childhood cancer according to whether IVF or ICSI was used as the method for fertilizing the oocyte, and whether the embryo was cryopreserved before embryo transfer. In Norway, the main indication for ICSI is male factor infertility, and ICSI is not commonly used for other indications such as total fertilization failure by IVF. Therefore, the specific increased cancer risk with ICSI could be potentially explained by factors associated with male factor infertility (the predominant underlying indication for ICSI), rather than mechanisms associated with ART use itself. However, as we also see differences with cryopreserved embryos, underlying uniparental subfertility is unlikely to be the only explanation for an increase in cancer risk in children conceived by ART. Disentangling the impact of ART treatment from the potential impact of the underlying parental cause of infertility is challenging, and this analysis was limited by an inability to take into account the indication for ART.

Additionally, the use of cryopreserved-thawed embryos was associated with a higher risk of childhood cancer compared to fresh embryo transfer. Trends over time show a shift towards more cycles performed by the use of cryopreserved embryos (Smeenk *et al.*, 2023), and more studies clarifying a potential cancer risk with this method are warranted. However, the maternal risk of hypertensive disorders of pregnancy is higher following the use of cryopreserved embryos, as is the risk of having a large-for-gestational-age or higher birth weight baby (Zaat *et al.*, 2021). Since higher birth weight is associated with childhood cancers including leukaemias, CNS tumours, and renal tumours (O’Neill *et al.*, 2015), this may partly explain some of our findings.

One potential mechanism associated with ART and cancer is epigenetic changes (Chen and Heilbronn, 2017). Epigenetic differences with ART conceptions have been found to mediate difference in birthweight associated with different ART methods (Carlsen *et al.*, 2022). It is currently uncertain how epigenetic findings in children conceived with ART specifically relate to future cancer outcomes for both boys and girls. Further research incorporating long-term follow-up is needed to investigate the long-term clinical significance of these epigenetic modifications in terms of cancer risk in both sexes.

### Strengths and limitations

This analysis is based on 39 years of high-quality Nordic registry data, including all live-born children born between 1984 and 2022. The Cancer Registry of Norway is regarded as having close-to-complete data on cancer diagnoses in Norway, with overall completeness estimated at 98.8% for the period 2001–2005 (Larsen *et al.*, 2009). Cancer diagnoses in childhood were classified according to ICCC-3 to enable comparability with previous studies. All clinics in Norway providing ART are required by law to report pregnancies conceived by ART to the MBRN, and this information is supplemented by a question about ART conception on the birth notification form. Information was not available for the indication for ART use during the whole study period, and it is possible that in a very small number of cases, the indication for ART use was for reasons other than subfertility, e.g. preimplantation genetic diagnosis.

Although we included all births from the year of the first ART birth in Norway, there were very few births following ART in the early years: 4% of ART births in our analysis occurred between 1984 and 1993 compared to 45% between 2014 and 2022. ART practices have changed immeasurably over this time period. To account for this, we adjusted for birth year and additionally conducted sensitivity analyses restricting to births from 2000 onwards, when both ICSI and the use of cryopreserved embryos were more prevalent.

Despite the large overall sample size, we were likely underpowered to look at age-specific risks and the risk associated with different ART methods (IVF vs ICSI, cryopreserved vs fresh embryo). Despite our overall finding of no significantly increased risk in either boys or girls, virtually all of the aHRs were suggestive of an increased risk (>1.00). Previous studies have found associations between ART conception and specific subtypes of childhood cancer such as hepatic cancer (Hargreave *et al.*, 2013; Williams *et al.*, 2013, 2018; Spector *et al.*, 2019; Weng *et al.*, 2022), neuroblastoma (Hargreave *et al.*, 2013), and retinoblastoma (Hargreave *et al.*, 2013; Lerner-Geva *et al.*, 2017), however, due to limited numbers in our study, we were generally unable to address associations for these rare subtypes.

We did not adjust for maternal smoking or BMI, as this information was not collected in the birth registry in earlier years. However, MBRN data from later years show that women conceiving via ART are less likely to smoke and did not significantly differ in BMI compared to the fertile population (Magnus *et al.*, 2021). Additionally, we did not have access to information on parental socioeconomic status. Although socioeconomic status is generally associated with ART use, the association is likely weaker in the Norwegian setting where most ART treatment is publicly funded. Additionally, there is scant evidence of a strong association with offspring risk of childhood cancer (Kehm *et al.*, 2018). We can thus assume any unmeasured confounding present would be unlikely to bias the true association.

## Conclusion

In conclusion, we did not observe strong evidence for an overall association between ART conception and childhood cancer before age 18 years in either boys or girls. However, the risk of being diagnosed with cancer between ages 5–9 years was increased in children conceived with ART, especially in boys. Conception via ICSI or following the use of cryopreserved embryos was associated with a higher risk of childhood cancer in boys. Additionally, there was evidence of an increased risk of leukaemia in ART-conceived boys. Our findings suggest that there may be sex differences in the impact of ART conception on childhood cancer risk driven by specific ART method. However, further research is needed, particularly given the continuing increase in the use of ICSI in cases without male factor infertility.

## Supplementary Material

deae285_Supplementary_Figure_S1

deae285_Supplementary_Figure_S2

deae285_Supplementary_Table_S1

deae285_Supplementary_Table_S2

deae285_Supplementary_Table_S3

deae285_Supplementary_Table_S4

deae285_Supplementary_Table_S5

deae285_Supplementary_Table_S6

deae285_Supplementary_Table_S7

## Data Availability

Study data are available on application via helsedata.no, subject to the necessary ethics approvals.
