# Supplementary material for: Sex differences in childhood cancer risk following ART conception: a registry-based study
Source: Hum Reprod. 2024 Dec 26;40(2):382–90. doi: 10.1093/humrep/deae285 (PMC11788205; doi:10.1093/humrep/deae285)
Supplement: deae285_Supplementary_Figure_S1 [file deae285_supplementary_figure_s1.pdf]

OXFORD
human reproduction

Human Reproduction, 2024, 1–1
https://doi.org/10.1093/humrep/deae285
Supplementary Data

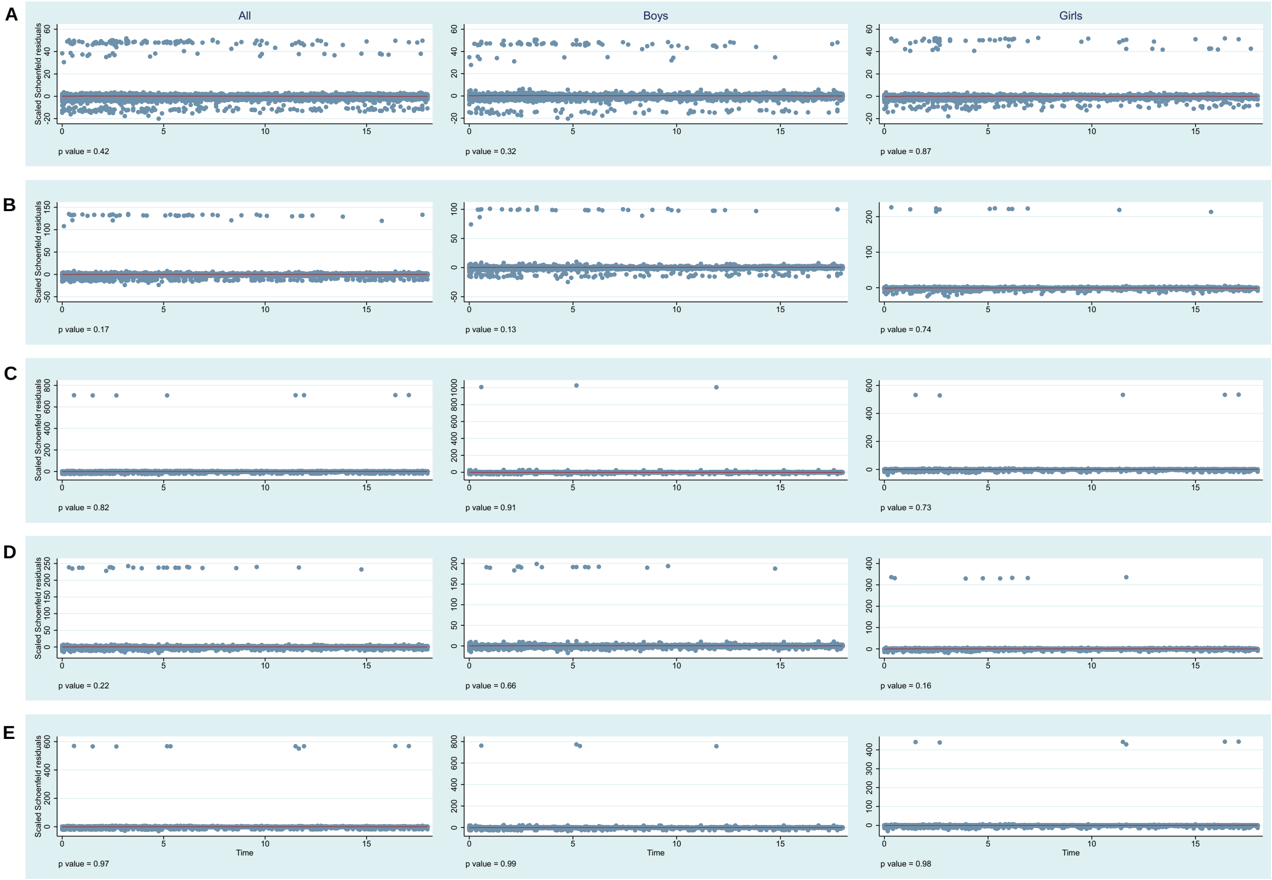

Supplementary Figure S1. Plots of scaled Schoenfeld residuals against time. (**A**) Any ART conception, (**B**) IVF conception, (**C**) ICSI conception, (**D**) fresh embryo, (**E**) cryopreserved embryo. P-values from null hypothesis of zero slope (Stata command ‘phtest’).
