# Supplementary material for: Sex differences in childhood cancer risk following ART conception: a registry-based study
Source: Hum Reprod. 2024 Dec 26;40(2):382–90. doi: 10.1093/humrep/deae285 (PMC11788205; doi:10.1093/humrep/deae285)
Supplement: deae285_Supplementary_Figure_S2 [file deae285_supplementary_figure_s2.pdf]

OXFORD
human reproduction
Human Reproduction, 2024, 1–1
https://doi.org/10.1093/humrep/deae285
Supplementary Data

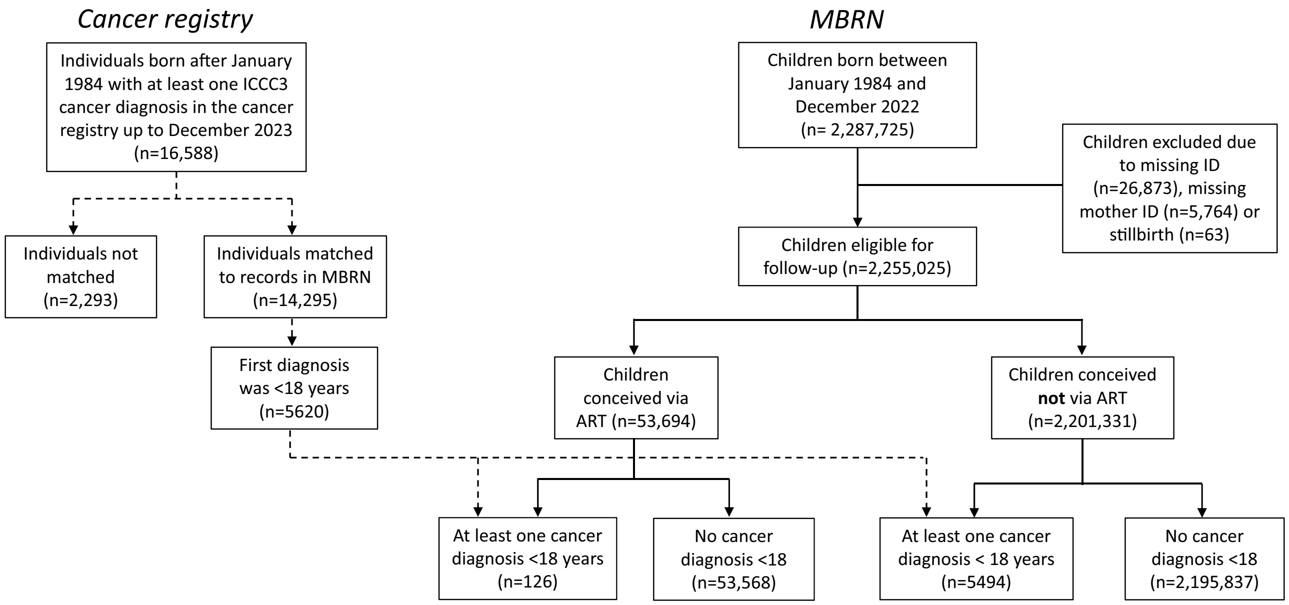

Supplementary Figure S2. Selection of eligible children. MBRN, Medical Birth Registry of Norway; ICCC3, International Classification of Childhood Cancer Third Edition.
