# Supplementary material for: Sex differences in childhood cancer risk following ART conception: a registry-based study
Source: Hum Reprod. 2024 Dec 26;40(2):382–90. doi: 10.1093/humrep/deae285 (PMC11788205; doi:10.1093/humrep/deae285)
Supplement: deae285_Supplementary_Table_S1 [file deae285_supplementary_table_s1.pdf]

Human Reproduction, 2024, 1–1
https://doi.org/10.1093/humrep/deae285
Supplementary Data

Supplementary Table S1. Description of data sources.

**Study population**	**Children**	The Medical Birth Registry of Norway[a]
**Study population**	**Deaths or emigration**	The National Registry (DSF)
**Exposure information**	**Conception method**	The Medical Birth Registry of Norway[b]
**Outcome information**	**Childhood cancer**	The Norwegian Cancer Registry
**Covariate information**	**Maternal age**<br>**Parity**<br>**Multiple birth**<br>**Maternal smoking**	The Medical Birth Registry of Norway
	**Parental history of cancer**	The Norwegian Cancer Registry

[a] Registry includes mandatory reporting of pregnancies in Norway ending after week 12.
[b] ART conception type recorded since the first ART-conceived pregnancy in Norway in 1984.
