# Supplementary material for: Sex differences in childhood cancer risk following ART conception: a registry-based study
Source: Hum Reprod. 2024 Dec 26;40(2):382–90. doi: 10.1093/humrep/deae285 (PMC11788205; doi:10.1093/humrep/deae285)
Supplement: deae285_Supplementary_Table_S2 [file deae285_supplementary_table_s2.pdf]

OXFORD

human reproduction

Human Reproduction, 2024, 1–1
https://doi.org/10.1093/humrep/deae285
Supplementary Data

Supplementary Table S2. Characteristics of ART conceptions.

ART method	Conceived via ART (IVF/ICSI) (N = 53 694)	
	n	(%)
**IVF or ICSI**		
IVF	28 851	(53.7)
ICSI	18 588	(34.6)
Combination/unknown/other	6255	(11.7)
**Fresh or cryopreserved embryo**		
Fresh	35 510	(66.1)
Cryopreserved-thawed	10 664	(19.9)
Unknown/other	7520	(14.0)

Note: presented as count (percentages).
