# Supplementary material for: Sex differences in childhood cancer risk following ART conception: a registry-based study
Source: Hum Reprod. 2024 Dec 26;40(2):382–90. doi: 10.1093/humrep/deae285 (PMC11788205; doi:10.1093/humrep/deae285)
Supplement: deae285_Supplementary_Table_S3 [file deae285_supplementary_table_s3.pdf]

OXFORD
human reproduction
Human Reproduction, 2024, 1–1
https://doi.org/10.1093/humrep/deae285
Supplementary Data

Supplementary Table S3. Overall and sex-stratified association between ART conception (IVF/ICSI) and childhood cancer <18 by ART method among births after 2000.

		All children			Boys			Girls		
		No. of cases	Hazard ratio (95% CI)		No. of cases	Hazard ratio (95% CI)		No. of cases	Hazard ratio (95% CI)	
			Unadjusted	Adjusted[a]		Unadjusted	Adjusted[a]		Unadjusted	Adjusted[a]
Any ART	Non-ART	2770	ref	ref	1477	ref	ref	1293	ref	ref
	ART (IVF/ICSI)	96	1.08 (0.88, 1.33)	1.06 (0.86, 1.32)	55	1.17 (0.89, 1.53)	1.14 (0.86, 1.52)	41	0.99 (0.73, 1.35)	0.97 (0.70, 1.34)
ART method	Non-ART	2770	ref	ref	1477	ref	ref	1293	ref	ref
	IVF	47	1.02 (0.77, 1.36)	1.01 (0.75, 1.36)	22	0.87 (0.57, 1.33)	0.86 (0.56, 1.33)	25	1.20 (0.81, 1.78)	1.19 (0.79, 1.77)
	ICSI	42	1.23 (0.91, 1.67)	1.20 (0.88, 1.64)	30	**1.72 (1.20, 2.47)**	**1.66 (1.14, 2.41)**	12	0.72 (0.41, 1.28)	0.71 (0.40, 1.26)
Embryo type	Non-ART	2770	ref	ref	1477	ref	ref	1293	ref	ref
	Fresh embryo	66	1.05 (0.82, 1.34)	1.04 (0.81, 1.34)	37	1.11 (0.80, 1.54)	1.09 (0.77, 1.54)	29	0.99 (0.68, 1.42)	0.98 (0.67, 1.42)
	Cryopreserved embryo	22	1.44 (0.94, 2.19)	1.38 (0.90, 2.11)	15	**1.81 (1.09, 3.00)**	**1.74 (1.04, 2.92)**	7	1.00 (0.47, 2.10)	0.95 (0.45, 2.01)

[a] Adjusted for birth year, maternal age, paternal age, multiple births, parity, and parental history of cancer.
The reference level corresponds to children conceived without ART. Bold font indicates statistical significance ($P < 0.05$).
