# Supplementary material for: Sex differences in childhood cancer risk following ART conception: a registry-based study
Source: Hum Reprod. 2024 Dec 26;40(2):382–90. doi: 10.1093/humrep/deae285 (PMC11788205; doi:10.1093/humrep/deae285)
Supplement: deae285_Supplementary_Table_S5 [file deae285_supplementary_table_s5.pdf]

OXFORD
human reproduction
Human Reproduction, 2024, 1–1
https://doi.org/10.1093/humrep/deae285
Supplementary Data

Supplementary Table S5. Bootstrap validation for overall and sex-stratified association between ART conception (IVF/ICSI) and childhood cancer by age group.

Age		ALL: Hazard ratio (95% CI) Adjusted[a]	ALL: Bootstrap validation Hazard ratio (95% CI) Adjusted[a]	BOYS: Hazard ratio (95% CI) Adjusted[a]	BOYS: Bootstrap validation Hazard ratio (95% CI) Adjusted[a]	GIRLS: Hazard ratio (95% CI) Adjusted[a]	GIRLS: Bootstrap validation Hazard ratio (95% CI) Adjusted[a]
**0 to <18 years**	**Non-ART**	ref	ref	ref	ref	ref	ref
	**ART (IVF/ICSI)**	1.13 (0.94, 1.36)	1.13 (0.94, 1.32)	1.22 (0.95, 1.57)	1.22 (0.97, 1.61)	1.03 (0.78, 1.37)	1.03 (0.77, 1.32)
**0 to <5 years**	**Non-ART**	ref	ref	ref	ref	ref	ref
	**ART (IVF/ICSI)**	1.09 (0.84, 1.42)	1.09 (0.81, 1.39)	1.23 (0.87, 1.74)	1.23 (0.84, 1.64)	0.95 (0.64, 1.41)	0.95 (0.60, 1.34)
**≥5 to <10 years**	**Non-ART**	ref	ref	ref	ref	ref	ref
	**ART (IVF/ICSI)**	**1.53 (1.06, 2.20)**	**1.53 (1.10, 2.18)**	**1.73 (1.09, 2.74)**	**1.73 (1.03, 2.57)**	1.28 (0.70, 2.33)	1.28 (0.60, 2.09)
**≥10 to <18 years**	**Non-ART**	ref	ref	ref	ref	ref	ref
	**ART (IVF/ICSI)**	0.91 (0.61, 1.34)	0.91 (0.64, 1.36)	0.79 (0.43, 1.44)	0.79 (0.39, 1.38)	1.02 (0.61, 1.71)	1.02 (0.63, 1.58)

[a] Adjusted for birth year, maternal age, paternal age, multiple births, parity, and parental history of cancer.
Note: The reference level corresponds to non-ART. The bootstrap method is a robust way to estimate the sampling distribution of a statistic, like HR, by repeatedly sampling from the dataset, particularly beneficial for small sample sizes. Bold font indicates statistical significance ($P < 0.05$).
