# Supplementary material for: Sex differences in childhood cancer risk following ART conception: a registry-based study
Source: Hum Reprod. 2024 Dec 26;40(2):382–90. doi: 10.1093/humrep/deae285 (PMC11788205; doi:10.1093/humrep/deae285)
Supplement: deae285_Supplementary_Table_S6 [file deae285_supplementary_table_s6.pdf]

Supplementary Table S6. Bootstrap validation for overall and sex-stratified association between ART conception (IVF/ICSI) and childhood cancer by ART method.

		ALL		BOYS		GIRLS	
		Hazard ratio (95% CI) Adjusted[a]	Bootstrap validation Hazard ratio (95% CI) Adjusted[a]	Hazard ratio (95% CI) Adjusted[a]	Bootstrap validation Hazard ratio (95% CI) Adjusted[a]	Hazard ratio (95% CI) Adjusted[a]	Bootstrap validation Hazard ratio (95% CI) Adjusted[a]
**ART method**	**Non-ART**	ref	ref	ref	ref	ref	ref
	**IVF**	1.18 (0.93, 1.49)	1.18 (0.96, 1.48)	1.07 (0.77, 1.51)	1.07 (0.81, 1.56)	1.29 (0.93, 1.80)	1.29 (0.84, 1.78)
	**ICSI**	1.18 (0.87, 1.59)	1.18 (0.86, 1.58)	**1.69 (1.18, 2.42)**	**1.69 (1.16, 2.46)**	0.65 (0.37, 1.16)	0.65 (0.33, 1.15)
**Embryo type**	**Non-ART**	ref	ref	ref	ref	ref	ref
	**ART—fresh embryo**	1.15 (0.93, 1.43)	1.15 (0.93, 1.41)	1.22 (0.91, 1.63)	1.22 (0.86, 1.66)	1.08 (0.78, 1.48)	1.08 (0.68, 1.43)
	**ART—cryopreserved embryo**	1.42 (0.95, 2.13)	1.42 (0.94, 2.06)	**1.79 (1.09, 2.94)**	**1.79 (1.16, 2.81)**	1.01 (0.50, 2.03)	1.01 (0.49, 1.89)

[a] Adjusted for birth year, maternal age, paternal age, multiple births, parity, and parental history of cancer.
Note: The reference level corresponds to non-ART. The bootstrap method is a robust way to estimate the sampling distribution of a statistic, like HR, by repeatedly sampling from the dataset, particularly beneficial for small sample sizes. Bold font indicates statistical significance ($P < 0.05$).

OXFORD
human reproduction
Human Reproduction, 2024, 1–1
https://doi.org/10.1093/humrep/deae285
Supplementary Data
