# Supplementary material for: Sex differences in childhood cancer risk following ART conception: a registry-based study
Source: Hum Reprod. 2024 Dec 26;40(2):382–90. doi: 10.1093/humrep/deae285 (PMC11788205; doi:10.1093/humrep/deae285)
Supplement: deae285_Supplementary_Table_S7 [file deae285_supplementary_table_s7.pdf]

OXFORD
human reproduction

**Supplementary Table S7.** Overall and sex-stratified association between ART conception (IVF/ICSI) and childhood cancer by childhood cancer type.

| ICCC3 cancer type (<18 years) | cases in ART (n) | All children Hazard ratio (95% CI) | | Boys Hazard ratio (95% CI) | | Girls Hazard ratio (95% CI) | |
|---|---|---|---|---|---|---|---|
| | | Unadjusted | Adjusted[a] | Unadjusted | Adjusted[a] | Unadjusted | Adjusted[a] |
| Leukaemia (I) | 44 | **1.43 (1.06, 1.93)** | 1.34 (0.97, 1.85) | **1.54 (1.04, 2.30)** | **1.60 (1.04, 2.46)** | 1.31 (0.83, 2.06) | 1.09 (0.67, 1.77) |
| Acute myeloid leukaemia (AML) | 9 | 1.78 (0.92, 3.46) | 1.79 (0.87, 3.69) | – | – | 2.72 (1.19, 6.18) | 2.16 (0.86, 5.45) |
| Acute lymphoid leukaemia (ALL) | 30 | 1.25 (0.87, 1.80) | 1.17 (0.79, 1.73) | 1.45 (0.91, 2.32) | 1.48 (0.89, 2.46) | 1.04 (0.59, 1.84) | 0.88 (0.48, 1.62) |
| Lymphomas (II) | 17 | 1.51 (0.93, 2.44) | 1.59 (0.96, 2.63) | 1.42 (0.76, 2.66) | 1.55 (0.80, 3.01) | 1.65 (0.78, 3.51) | 1.65 (0.77, 3.56) |
| Central nervous system tumours (III) | 32 | 1.08 (0.76, 1.53) | 0.97 (0.67, 1.41) | 0.94 (0.56, 1.56) | 0.80 (0.46, 1.39) | 1.24 (0.77, 2.01) | 1.19 (0.72, 1.96) |
| Neuroblastoma and other peripheral nervous cell tumours (IV) | 5 | 0.80 (0.33, 1.94) | 0.80 (0.34, 1.89) | – | – | – | – |
| Retinoblastoma (V) | <5 | – | – | – | – | – | – |
| Renal tumours (VI) | 8 | 1.64 (0.81, 3.31) | 1.77 (0.85, 3.69) | 2.38 (0.97, 5.84) | 2.34 (0.86, 6.38) | – | – |
| Hepatic tumours (VII) | <5 | – | – | – | – | – | – |
| Bone tumours (VIII) | <5 | – | – | – | – | – | – |
| Soft tissue sarcomas (IX) | 8 | 1.46 (0.73, 2.95) | 1.31 (0.61, 2.83) | **2.22 (1.04, 4.72)** | 2.16 (0.93, 5.02) | – | – |
| Germ cell and gonadal tumours (X) | <5 | – | – | – | – | – | – |
| Epithelial tumours and melanoma (XI) | <5 | – | – | – | – | – | – |
| Other and unspecified tumours (XII) | <5 | – | – | – | – | – | – |

[a] Adjusted for birth year, maternal age, paternal age, multiple births, parity, and parental history of cancer.
All models include robust standard errors to account for dependency between siblings. Bold font indicates statistical significance ($P < 0.05$).

Human Reproduction, 2024, 1–1
https://doi.org/10.1093/humrep/deae285
Supplementary Data
